# Near-zero temperature coefficient of resistance of hybrid resistor fabricated with carbon nanotube and metal alloy

**DOI:** 10.1038/s41598-019-44182-7

**Published:** 2019-05-23

**Authors:** Sunwoo Lee, Eun-Min Kim, Youngtaek Lim

**Affiliations:** 1 Department of Electrical Information, Inha Technical College, Incheon, 22212 Korea; 2 Electric Power Research Institute, Seoul National University, Seoul, 08826 Korea

**Keywords:** Sensors and biosensors, Electrical and electronic engineering

## Abstract

A hybrid resistor has been fabricated by parallely connecting carbon nanotube (CNT) fiber with negative temperature coefficient of resistance (TCR) and metal alloy with positive TCR to achieve near zero TCR. The CNT fibers were prepared by yarning CNTs grown on the silicone substrate by chemical vapor deposition (CVD) method. The CNT fiber resistors were fabricated by winding CNT fiber on the ceramic rod. Metal terminals were connected at both ends of the CNT fiber wound on the ceramic rod. The metal alloy resistors were fabricated with copper (Cu) and nickel (Ni) with different weight compositions. Electrical resistance and thermal stability (in terms of TCR in this work) of the CNT fiber resistors, the metal alloy resistors, and the hybrid resistors were measured as 7.94 Ω and −870 ppm/°C, 7.94 Ω and 1100 ppm/°C, and 3.97 Ω and −2 ppm/°C, respectively. In case of parallelly connected resistors with suitable combination, the resistance was lower than that of resistor with lower value, and the TCR approached to near zero. Finally, we propose a theoretical approach for adjusting resistance and TCR of the hybrid resistor composed of metal alloy and CNT fibers.

## Introduction

As IT technologies advances, network based portable devices, sensors and secondary batteries have attracted great attention in the worldwide market^1^. Secondary batteries could last for a long period by repetitive charging and discharging processes. During this charging and discharging process, battery management system (BMS) is necessary for controlling the charging and discharging process effectively by distributing source charges among battery cells. To control the charge distribution among battery cells, current sensing resistor (CSR) has been used for measuring current in the BMS^2^. This technique is the conventional way of sensing the electric power by inserting a CSR in series (constant voltage and variable current, consequently variable product power). With known resistance of the resistor, the current flowing through the resistor is determined by sensing the voltage drop across it^3^. In the case of varied resistance value at the varied temperature, the CSR shows inaccurate sensing result for the varied resistance resulting in severe failure such as over-charging, explosion and so on^4^. Therefore, fixed resistance with temperature is strongly required for the stable current sensing characteristics. As common CSRs are made of metal alloy, resistance of the CSR increases with the temperature resulting in sensing error at the elevated temperature. In industry, it is a common practice to reduce the temperature coefficient of resistance (TCR) by alloying metals with low TCR material such as manganease or nickel sacrificing resistance or less resistive metals such as copper with high TCR^5^. However using these approaches with trade-off relation, they could not obtain the zero TCR resistors, but low TCR resistors with about 20 (at the laboratory level) to 50 ppm/°C (at the mass-production level)^6,7^. Therefore, they have not realize the zero TCR resistors without sensing errors yet.

Due to the fact that carbon nanotube (CNT) is composed of a hexagonal carbon structure having strong carbon double bonds, the electromigration phenomenon is expected to be negligible^8^. CNT has a nearly perfect one-dimensional structure, and thus, it has been pointed out that CNT could exhibit ballistic transport without lattice or grain boundary scattering^9,10^. This is the reason CNT is attracting much interest as a candidate for conducting materials. Moreover, CNT shows semiconducting nature on average. In the CNTs grown by chemical vapor deposition (CVD) method, about one-third of the CNTs shows metallic nature and two-third of them show semiconducting nature^11–14^. Therefore average electrical nature of the CNTs grown by CVD method is expected to be semiconducting property resulting in negative TCR^15–17^.

In the present work, we propose a novel approach to fabricate the hybrid type resistor with near zero TCR composing metal alloy and carbon nanotube (CNT) fiber for use of the CSRs.

## Experimental

The CNT fibers were prepared by yarning CNTs grown on the silicon wafer by CVD method. Grown CNTs were multi-walled carbon nanotubes and their morphology was investigated by using scanning electron spectroscope (SEM) and transmission electron spectroscope (TEM). The CNT fiber resistors used in this work were fabricated by winding CNT fibers on the ceramic rod. Metal terminals were connected at both ends of the CNT fiber wound on the ceramic rod. Electrical properties such as resistance and TCR of the CNT resistors were measured.

The metal alloy resistors were fabricated by alloying copper (Cu) and nickel (Ni) with different atomic compositions designed to adjust resistance and TCR of them to those of the CNT fiber resistors fabricated in this work. Each ingot typed metal alloy was fabricated by melting the designed amount of Ni and Cu in the vacuum melting furnace. Each ingot fabricated was annealed for uniform dispersion. And the concentration was adjusted by segregation elimination method. Finally, metal alloy wires were fabricated by hot rolling method. Atomic compositions of the metal alloy wires fabricated were confirmed by using the energy dispersive X-ray analysis (EDX) for confirming the composition design result.

**Figure 1:**
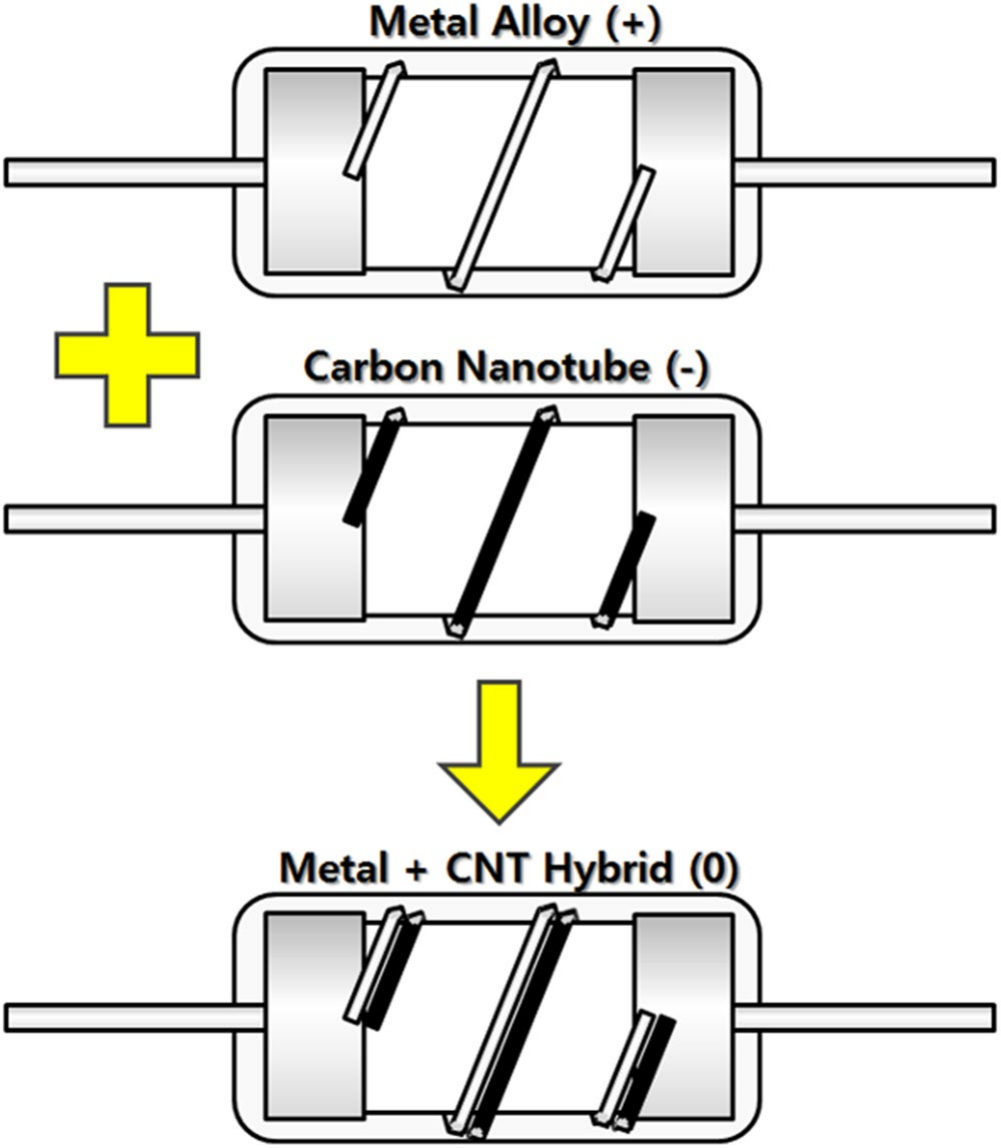
Schematic of the hybrid resistor designed in this work.

The hybrid resistors were fabricated by connecting above two kinds of resistors fabricated (CNT fiber resistor and metal alloy resistor) parallelly, and schematic of the hybrid resistor was shown in Fig. 1. First, metal alloy wire was wound on the resistor body, which composed of ceramic rod, metal caps, and lead wires. And then, both ends of the wire were fixed and electrically connected by spot welding method. Finally, CNT fibers were wound on the resistor body and fixed and electrically connected by pasting with silver paste. Electrical properties were also measured in terms of resistance and TCR. TCR measurement was done by measuring resistance from 25 to 125 °C with 5 °C step raising in the vacuum furnace.

## Results and Discussions

**Figure 2:**
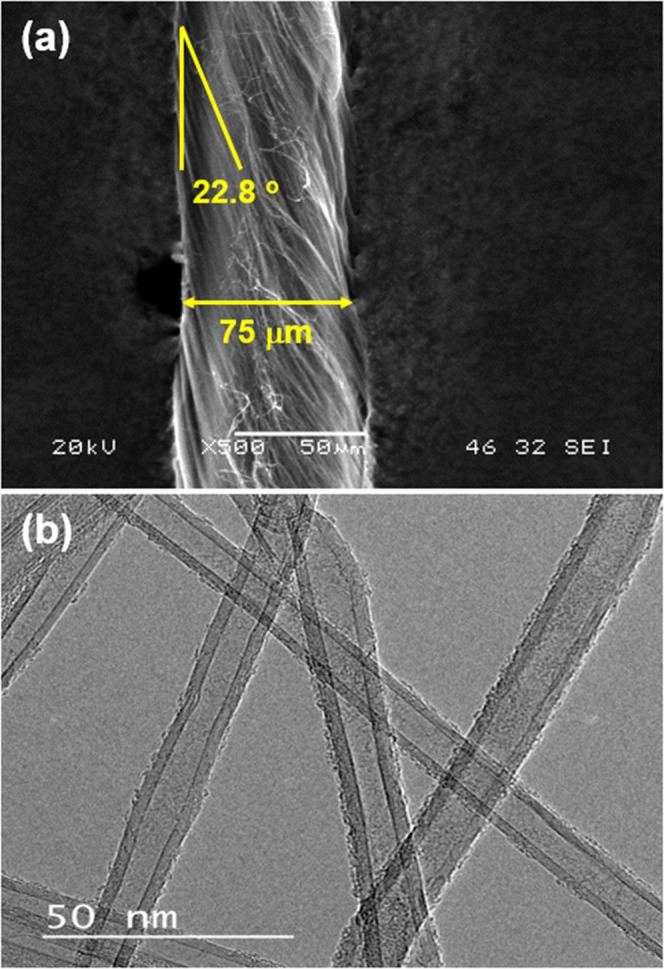
(**a**) SEM image and (**b**) TEM image of the CNT fiber used in this work.

One thread of CNT fiber used in this work show 75 μm of diameter and 22.8 degree of twist angle as shown in Fig. 2(a). The CNT fiber is composed of many threads of multi-walled CNT (MWCNT) as shown in Fig. 2(b).

**Figure 3:**
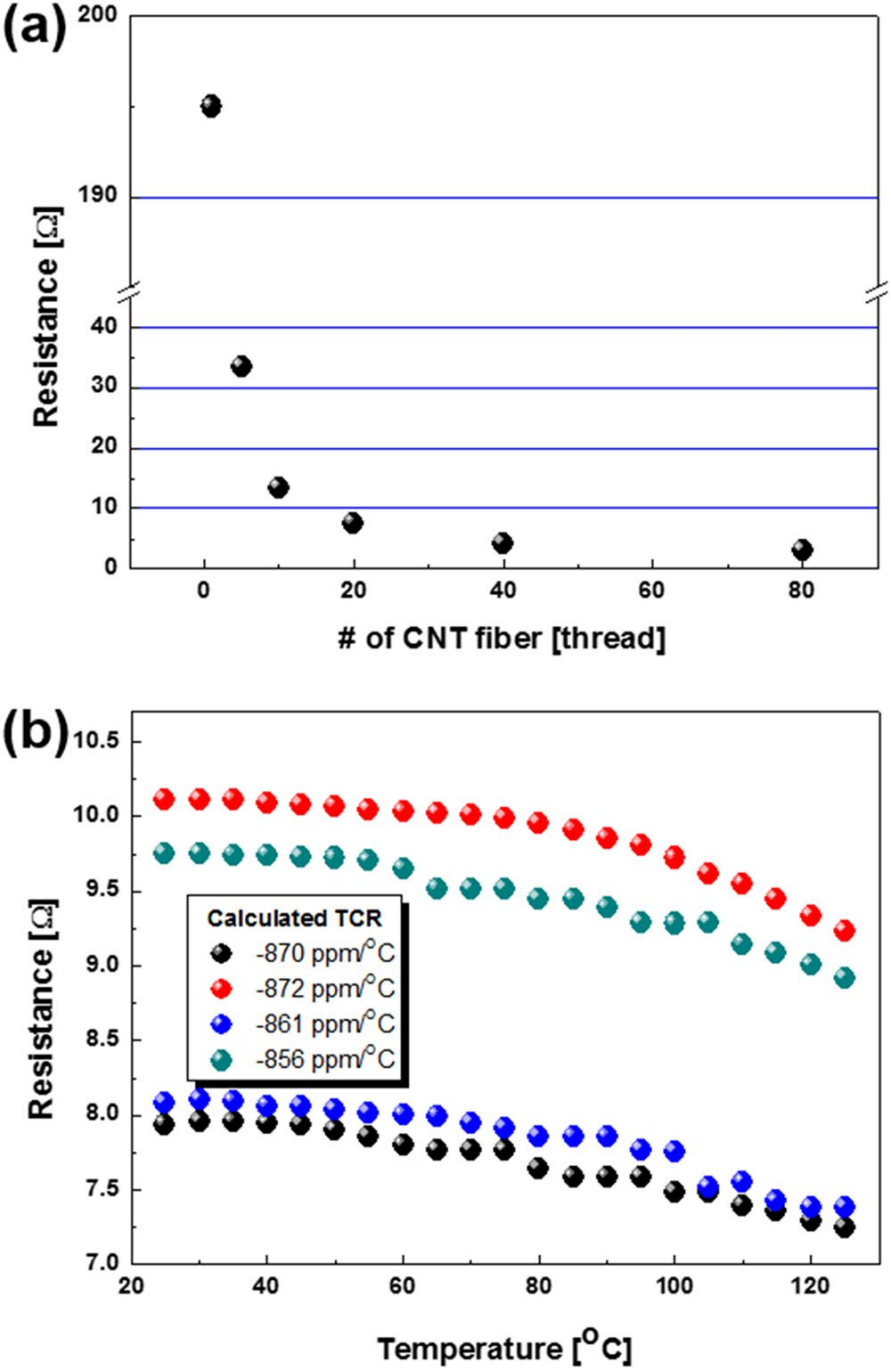
Electrical properties of the CNT fibers; (**a**) resistances with the number of threads and (**b**) TCR of the CNT fibers.

Electrical properties of the CNT fibers were measured and showed in Fig. 3. Resistance of the CNT fibers decreased with increasing the number of threads as shown in Fig. 3(a). Four CNT fiber resistors were fabricated by twisting the 10 threads of CNT fibers, and showed resistance values around 8 to 10 Ω as shown in Fig. 3(b).

TCRs of the four CNT fiber resistors were calculated by resistance change over temperature change from 25 to 125 °C by using the Eq. (1), and were showed in inset of the Fig. 3(b). Here, R_1_ and R_2_ are resistances at temperatures of T_1_ and T_2_, respectively^18^.

1 $$TCR=\frac{{R}_{2}-{R}_{1}}{{R}_{1}({T}_{2}-{T}_{1})}\times {10}^{6}\,[ppm/{}^{\circ }{\rm{C}}]$$

Although the resistance of the CNT fibers decreased with the increasing the number of threads above mentioned, the TCR of them was fixed between −800 to −900 ppm/°C as shown in Fig. 3(b). The CNT fiber showed negative TCR for its semiconducting nature on average. In the CNTs grown by CVD method, about one-third of the CNTs shows metallic nature and two-third of them show semiconducting nature^19^. Therefore average electrical nature of the CNTs grown by CVD method becomes semiconducting property resulting in negative TCR^20^.

In order to meet the requirements of the resistor such as resistance and TCR, the metal alloy resistors were designed with fixed resistance and same but positive TCR with that of the CNT fiber resistors. Low and fixed resistance could be served by Cu and the TCR could be adjusted by adding Ni with different weight percent in the alloy. The amount of Ni added in the alloy was varied to adjust the TCR of the metal alloy resistors between 400 to 1300 ppm/°C. And the resistance values of the metal alloy resistors were adjusted by changing length of them. Finally, we prepared four sets of metal alloy resistors with various TCRs, as shown in Table 1.

**Table 1:** Atomic composition versus electrical properties of the metal alloy resistors.

Atomic composition	Electrical properties of the metal alloy resistor sets for the CNT samples (R[Ω], TCR[ppm/°C])			
	CNT Sample #1	CNT Sample #2	CNT Sample #3	CNT Sample #4
Cu-35Ni	7.94, 400	10.11, 400	8.08, 400	9.75, 400
Cu-12Ni	7.94, 700	10.11, 700	8.08, 700	9.75, 700
Cu-9Ni	7.94, 800	10.11, 800	8.08, 800	9.75, 800
Cu-8Ni	7.94, 900	10.11, 900	8.08, 900	9.75, 900
Cu-5Ni	7.94, 1100	10.11, 1100	8.08, 1100	9.75, 1100
Cu-4Ni	7.94, 1300	10.11, 1300	8.08, 1300	9.75, 1300

**Figure 4:**
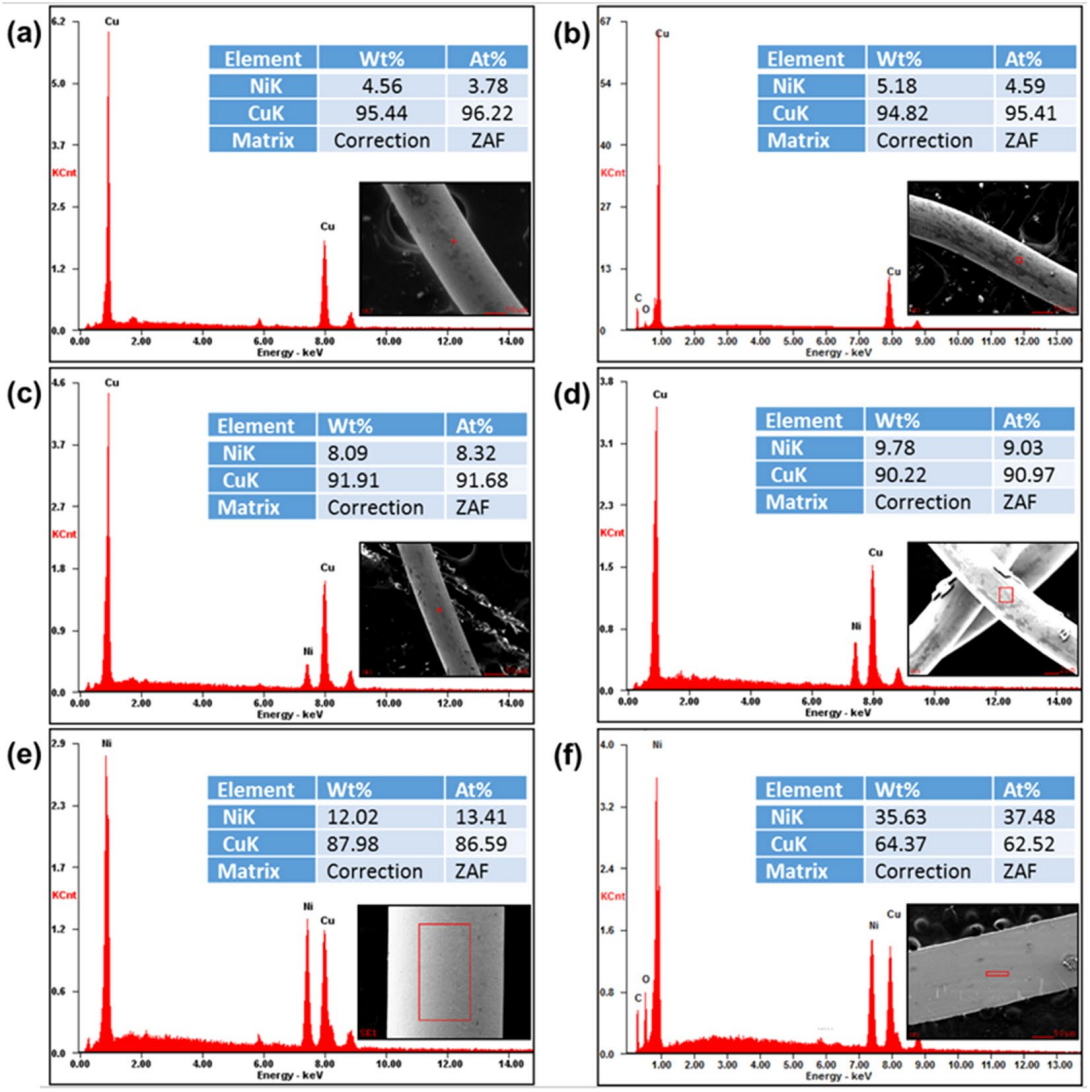
Atomic composition of the metal alloy resistor samples measured by EDX; (**a**) Cu-35Ni, (**b**) Cu-12Ni, (**c**) Cu-9Ni, (**d**) Cu-8Ni, (**e**) Cu-5Ni, and (**f**) Cu-4Ni samples.

Atomic compositions of the metal alloy resistor samples were confirmed by the EDX, and the results were shown in Fig. 4. The EDX results show that metal alloys were successfully prepared as designed. Manganese (Mn) as microelement detected in some samples was contaminant contained in copper (Cu), but it did not show any significant effect on the electrical properties of the metal alloy resistors.

**Figure 5:**
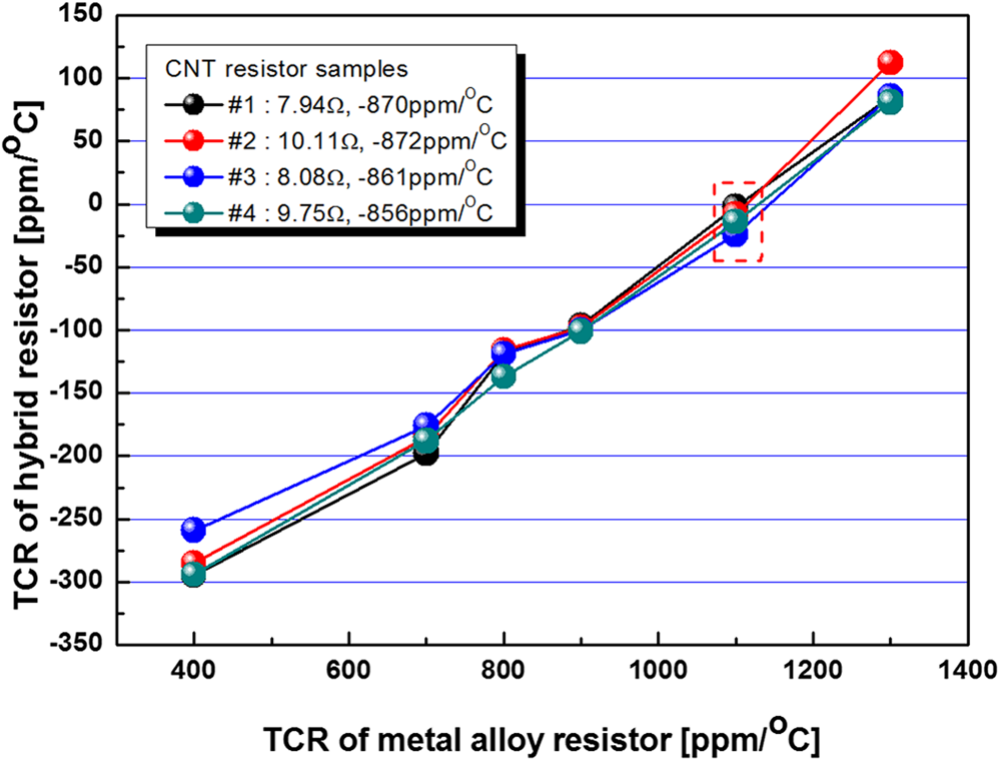
TCRs of the hybrid resistor combinations of four CNT resistors and six metal alloy resistors.

Hybrid resistors composed of the CNT fiber and the metal alloy were fabricated by connecting them parallelly. Four CNT resistor samples with negative TCR were connected parallelly with six metal alloy resistors with same resistance with positive TCRs between 400 to 1300 ppm/°C, resulting in 24 combinations of hybrid resistors. Resistance of the hybrid resistor was half of the CNT or metal alloy resistors with same resistance, because they were connected parallelly. And the TCR of the hybrid resistors were measured and shown in Fig. 5. The TCR of the hybrid resistor increased linearly with the TCR of the metal alloy resistor. We expected that the TCR of the hybrid resistor would show near zero TCR at the combination of same but opposite sign of TCRs; −865 ppm/°C (average value) for the CNT fiber resistor and +865 ppm/°C for the metal alloy resistor. In the actual case of the CNT resistor sample #1 with 7.94 Ω and −870 ppm/°C, expected resistance increase for the metal alloy resistor by increasing temperature from 25 to 125 °C would be 0.69 Ω, and expected resistance decrease for the CNT fiber resistor by increasing temperature from 25 to 125 °C would be 0.69 Ω. Therefore, resistance change should be compensated to be zero by connecting them parallelly. However, the hybrid resistors actually showed the near zero TCR at around 1100 ppm/°C for the metal alloy resistors. The hybrid resistors composed of the CNT fiber with −865 ppm/°C and the metal alloy with 1100 ppm/°C showed −2 ppm/°C for the CNT resistor sample #1, −8 ppm/°C for the CNT resistor sample #2, −24 ppm/°C for the CNT resistor sample #3, and −14 ppm/°C for the CNT resistor sample #4, respectively. This result might be explained from errors during measurement; lead wire has completely different resistance and TCR with the CNT fiber resistors or metal alloy resistors.

**Figure 6:**
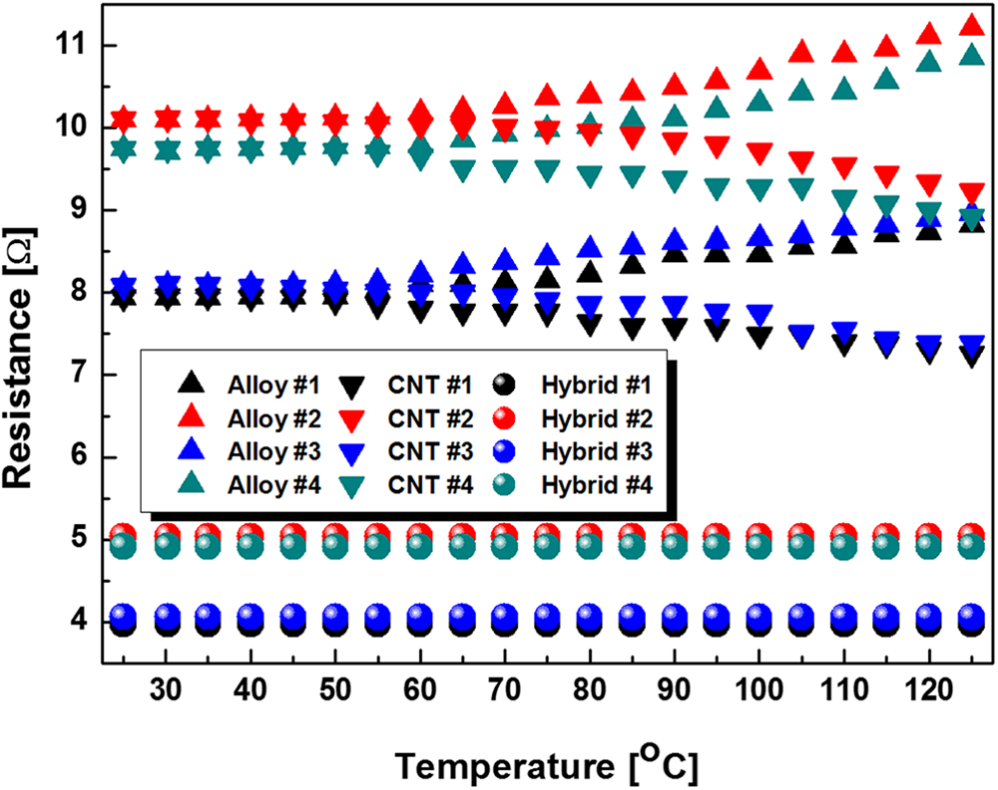
Resistance of the hybrid resistors showing the near zero TCR.

Finally, we measured resistance of the metal alloy, the CNT fiber, and the hybrid resistor showing the near zero TCR by step-wisely with the temperature step of 5 °C as shown in Fig. 6. As shown in the Fig. 6, TCR of the metal alloy resistor (upper triangular symbols) increased with the temperature, resulting in positive TCR. However, TCR of the CNT fiber resistor (lower triangular symbols) decreased with the temperature, resulting in negative TCR. Therefore, the hybrid resistor composed of the metal alloy resistor and the CNT fiber resistor showed near zero TCR with thermal stability.

## Conclusion

We proposed a novel approach for fabricating resistors with near zero TCR by parallelly connecting CNT fiber with negative TCR and metal alloy with positive TCR. The CNT fiber resistors fabricated by winding CNT fibers showed −870 ppm/°C of TCR. The metal alloy resistors were fabricated with Cu and Ni with different weight compositions showing TCR between 400 to 1300 ppm/°C. The hybrid resistors fabricated by connecting above two resistors parallelly showed near zero TCR of −2 ppm/°C. Resistance increase at the metal alloy resistor and resistance decrease from the CNT fiber resistor by increasing temperature were compensated each other, resulting in near zero TCR at the hybrid resistor. Finally we fabricated resistors with near zero TCR by connecting the metal alloy resistor with positive TCR and the CNT fiber resistor with negative TCR parallelly.
